# Miniaturized-LC in the Analysis of Emerging Organic Contaminants in Food and Environmental Samples: Recent Advances and Applications

**DOI:** 10.3390/molecules31010068

**Published:** 2025-12-24

**Authors:** Cemil Aydoğan, Ashraf Ali, Mehmet Atakay, Bekir Salih, Ziad El Rassi

**Affiliations:** 1 Food Analysis and Research Laboratory, Bingöl University, 12000 Bingöl, Türkiye; 2 Department of Chemistry, Bingöl University, 12000 Bingöl, Türkiye; 3 Department of Food Engineering, Bingöl University, 12000 Bingöl, Türkiye; 4 School of Chemistry and Chemical Engineering, Henan University of Technology, Zhengzhou 450000, China; ashrafchemist12@gmail.com; 5 Department of Chemistry, Hacettepe University, 06800 Ankara, Türkiye; mehmetatakay@hacettepe.edu.tr (M.A.); bekir@hacettepe.edu.tr (B.S.); 6 Turkish Academy of Sciences, 06670 Ankara, Türkiye; 7 Department of Chemistry, Oklahoma State University, Stillwater, OK 74078-3071, USA

**Keywords:** Capillary-LC, Chip-LC, emerging organic contaminants (EOCs), mycotoxins, Nano-LC, pesticides, veterinary drugs

## Abstract

Mini-LC systems, including Cap-LC, Nano-LC and Chip-LC, offer a sustainable alternative to conventional LC methods thanks to their reduced solvent consumption, enhanced separation efficiency and environmentally friendly operation. Integrating micro-scale sample preparation techniques, such as µ-SPE, IT-SPME, LPME and QuEChERS, with Mini-LC significantly improving analytical sensitivity and selectivity. Mini-LC coupled with mass spectrometry has demonstrated excellent performance in the detection of trace levels of pesticides, pharmaceuticals, veterinary drug residues, perfluoroalkyl substances (PFASs), and mycotoxins. Despite current challenges relating to matrix effects, instrument stability and method standardization, Mini-LC represents a promising analytical platform for the cost-effective, high-sensitivity, green monitoring of contaminants in food safety and environmental analysis. This review summarizes recent advances in the application of Mini-LC techniques for analyzing emerging organic contaminants (EOCs) in food and environmental samples. This paper also provides a critical review of this topic, covering works published in the last four years (early 2022–mid 2025). Additionally, it discusses the use of these techniques in combination with mass spectrometry (e.g., low-resolution MS or high-resolution MS) for the detection of EOCs in food and environmental samples.

## 1. Introduction

Miniaturized-LC (Mini-LC) systems have attracted increasing attention due to their potentials as advanced chromatographic separation processes. These highly adaptable platforms allow for the sensitive analysis of a variety of compounds [1]. When used with mass spectrometry, these systems greatly enhance analyte ionization efficiency. Mini-LC is widely used in several fields including proteomic and metabolomic mixtures of new chemical entities [2,3,4], food safety [5,6], pharmaceuticals [7], molecular toxicology [8] and drug discovery [9]. One of the most notable advantages of Mini-LC is the reduced consumption of chemicals, which not only lowers costs, but also minimizes environmental impact. In the context of Mini-LC systems, narrow columns with an internal diameter (ID) of less than 1 mm are used, which significantly reduces injection volumes and flow rates. Different types of columns are used in Mini-LC, such as particle-packed [10], monolithic columns [7] and open tubular columns [11]. Micro-pillar array columns and chip columns are also used in Mini-LC or portable-LC systems [12]. Although particle-packed columns are widely used in Mini-LC, monolithic columns are a promising alternative material. Monolithic columns have several features, such as lower back pressure and high versatility [13]. These narrow bore columns offer several notable benefits, including reduced chromatographic dilution, lower solvent consumption, and greater efficiency. However, these benefits come with challenges, such as the fact that the dead volume becomes more significant at reduced scales, potentially leading to band broadening. Researchers have addressed this issue by using connecting tubing with very small inner diameter and zero dead volume fittings to minimize the overall dead volume of the setup [14].

Mini-LC techniques are also a rapidly emerging tool for analyzing organic contaminants and detecting low-abundance contaminants such as organic pollutants in different samples that require higher sensitivity. Recently, the importance of EOCs has grown significantly, mainly due to advancements in miniaturized analytical systems that allow for their detection at high sensitivity, typically ranging from nanograms to micrograms per liter. Research has shown that surface waters and wastewater contain endocrine-disrupting compounds (EDCs), such as hormones and pharmaceuticals. This illustrates the inefficiency of traditional wastewater treatment methods in eliminating these pollutants. Thereupon, researchers and public health advocates are increasingly concerned about these pollutants because they can accumulate in nature, persist in the environment for extended periods, and cause harmful effects.

The analysis of organic pollutants is a key issue in environmental analytical chemistry. The production and consumption of harmful chemicals have resulted in thousands of compounds being discharged into the environment. The high persistence and mobility of some of these substances causes them to migrate away from the site of application, reaching surface and groundwater, and consequently bioaccumulating in the food chain [15]. The EU Water Framework Directive established controls on a list of persistent chemicals in the environment that can bioaccumulate and pose a risk to human health and the environment [16]. These regulated compounds are controlled and monitored worldwide using analytical methods. Many substances classified as priority pollutants are pesticides, such as organochlorides, organophosphates, triazines and sulfonylureas. There are several regulations concerning the application of pesticides and concentration limits in the environment and in drinking water [17,18]. The legal limit for individual pesticides in drinking water is generally 0.1 µg L^−1^, while the limit for total pesticides is 0.5 µg L^−1^. For surface waters, the environmental quality standard varies for each pesticide, ranging from ng L^−1^ to µg L^−1^ [19]. EOCs, which include pharmaceuticals, personal care products, pesticides, plasticizers and industrial additives, are increasingly being detected at trace levels in food and environmental matrices, posing risks to both the environment and human health. For example, antibiotic residues in dairy products and aquaculture environments have been linked to antimicrobial resistance, and persistent plasticizers such as bisphenol A and phthalates have been shown to disrupt the endocrine system in wildlife and humans. Furthermore, trace-level herbicides and pharmaceutical metabolites found in surface water can bio-accumulate through the food chain, thereby adversely affecting aquatic ecosystems and entering the food supply. These findings highlight the urgent need for robust, sensitive and sustainable analytical strategies to monitor and mitigate EOCs in complex matrices. In this context, Mini-LC technologies offer significant advantages by combining high separation efficiency with reduced solvent consumption and enhanced portability. This makes them particularly suitable for on-site, high-throughput analysis of EOCs in food safety and environmental assessment.

Miniaturization and automation are becoming an ever-popular necessity in modern chromatography [20]. In this sense, Mini-LC is achieved through reduction in the analytical column internal diameter, which is directly related to the system flow rate [21]. The development of portable LC instruments and on-line sample processing methods that make in situ analysis feasible has significantly contributed to the advanced organic contaminant analysis in both environmental monitoring and other fields. This review article summarizes the fundamental principles of Mini-LC systems, discusses technological developments and analysis strategies, and reports on some recent applications involving mass spectrometry (e.g., low-resolution and high-resolution MS) from 2022 to the present day.

## 2. Emerging Organic Contaminants

Emerging organic contaminants are substances that can be present in certain foodstuffs or different samples due to environmental contamination, production processes, cultivation practices, or environmental contamination. Recently, in response to regulated contaminants (pesticides, veterinary drugs, toxins, food and plant contaminants, etc.) several substances have emerged as EOCs, which can be defined as unregulated and recently discovered potentially harmful to human health. These EOCs have become a hot topic in the critical issue of 21st-century food and environmental safety, which poses a serious threat to human health and life worldwide [22] due to their toxicity mechanisms not being fully understood. Legislation is being revised to include new information that had not previously been taken into account, and tested methodologies have improved [23]. Recent studies have investigated certain types of EOCs, looking into where they come from, how they get there, and what they do. Agricultural runoff often contains herbicides and pesticides that pollute nearby bodies of water [24,25]. Studies have shown how EOCs impact aquatic organisms, complicating the ecosystems they invade [26]. Furthermore, when environmentally relevant contaminants interact with other pollutants, their combined effects can intensify both ecological damage and toxicity, resulting in a greater overall impact than either substance alone [27].

Addressing the challenges related to EOCs requires a multidisciplinary approach that unites expertise in toxicology, environmental science, and analytical chemistry. New methods for treating water, such as advanced oxidation processes and biofiltration, are being explored to determine their effectiveness in removing EOCs. At the same time, better ways to monitor water quality are being developed to detect EOCs more effectively [28]. It is crucial for regulatory agencies, businesses, and research institutions to collaborate in establishing rules for managing and mitigating the risks associated with EOCs. The challenge posed by EOCs necessitates an ongoing commitment to research and innovation in both analysis and remediation strategies. The chemical complexity of EOCs requires analytical platforms that can deliver high sensitivity and resolution, as well as the ability to quickly handle low sample volumes. A major area of research is finding better ways to analyze these species, such as using Mini-LC techniques to improve the sensitivity and specificity of detection. Mini-LC systems are a useful alternative to traditional LC systems as they are easier to transport, use less solvent and can achieve high-efficiency separations in a more compact format.

Mini-LC techniques are becoming increasingly important for determining contaminants because they transfer mass more efficiently, broaden signal peaks less and are compatible with low-volume, high-throughput workflows. This is especially useful given that emerging organic pollutants are usually found in very small amounts, in complicated matrices and in a wide range of structures. Not only is Mini-LC a useful addition to existing methods, it is also a necessary step forward in solving current analytical problems in EOC analysis.

## 3. Sample Preparation for the Determination of EOCs in Mini-LC

The analysis of EOCs such as pharmaceuticals, personal care products, pesticides, and industrial chemicals is notoriously challenging due to their low concentrations in complex environmental, food, and biological matrices [29]. The transition from conventional HPLC to Mini-LC systems encompassing capillary-LC (Cap-LC), Nano-LC and Chip-LC presents a paradigm shift in analytical science, offering enhanced mass sensitivity, reduced solvent consumption, and superior compatibility with mass spectrometry (MS) [30]. However, this miniaturization places stringent demands on the sample introduction process. The core challenge lies in the volumetric mismatch between typical sample preparation volumes (microliters) and the nanoliter-scale injection volumes required to maintain chromatographic efficiency in Mini-LC [31]. Traditional sample preparation methods are not well-suited for Mini-LC, as they can require large solvent volumes and introduce significant dead volume, negating the sensitivity benefits of miniaturization. The field has therefore shifted towards developing miniaturized, selective, and efficient sample preparation strategies that are seamlessly compatible with Mini-LC systems [32]. Sample preparation is a critical step in Mini-LC analysis of EOCs, as the reduced column dimensions and low flow rates necessitate highly efficient extraction and pre-concentration techniques to achieve optimal sensitivity and reproducibility [1]. Due to the trace-level concentrations of EOCs in complex matrices (e.g., environmental waters, biological fluids, and food samples), effective sample cleanup and enrichment are essential [33]. Solid-phase extraction (SPE) remains the most widely used method, offering high selectivity through various sorbents (e.g., C18, hydrophilic-lipophilic balance, molecularly imprinted polymers (MIPs), and mixed-mode phases) tailored for specific contaminant classes such as pharmaceuticals, pesticides, or PFAS [34]. For even greater sensitivity, miniaturized SPE (µ-SPE) and dispersive SPE (d-SPE) reduce solvent consumption and improve extraction efficiency [35].

Recently, Andreasidou et al. [36] developed an acrylic copolymer-based dispersive solid-phase microextraction (d-µ-SPE) method for extracting 24 endocrine-disrupting chemicals (EDCs) from wastewater, followed by liquid chromatography-mass spectrometry (LC-MS) analysis. This method has a low LOD (2–92 ng L^−1^) and LOQ (6–279 ng L^−1^), and a wide linear range of 100–33,300 ng L^−1^. Liquid-phase microextraction (LPME) methods, including dispersive liquid–liquid microextraction (DLLME) and hollow-fiber LPME (HF-LPME), provide significant enrichment factors while reducing co-extracted matrix interferences. Recently, Domínguez-Liste et al. [37] developed a liquid–liquid extraction (LLE) method for extracting endocrine-disrupting chemicals from semen, followed by LC-MS/MS analysis. This method showed a low LOD of 0.02–0.3 ng mL^−1^ and good recovery rates of 85.5–112.5%. PFAS and phenolic EDCs were quantified in serum samples from 21 volunteers. QuEChERS is commonly used to analyze multi-residue analytes in different samples, and it can be combined with µ-SPE or online cleanup to produce extracts suitable for Mini-LC. Nguyen and Baduel [38] developed a QuEChERS-based UPLC-MS/MS method for extracting, purifying and analyzing 90 EOCs, including pharmaceuticals, flame retardants, plasticisers and PFAS, in soil and sediment. The developed method demonstrated good accuracy, precision, and recovery (70–120% with RSD <20%), as well as good linearity, low matrix effects, and low LOQs (0.25–10 µg kg^−1^). More details on the comparison of miniaturized sample preparation techniques for mini-LC analysis of EOCs were given in Table S1.

Recent advancements in on-line sample preparation, such as in-tube solid-phase microextraction (IT-SPME) and turbulent flow chromatography (TFC), enable direct coupling with Mini-LC systems, enhancing automation and reducing analyte loss [39]. Despite these innovations, challenges remain, including matrix effects, the need for stringent method optimization, and the limited loading capacity of Mini-LC columns [12]. The success of any Mini-LC analysis hinges on a robust and efficient sample introduction (i.e., injection) system. The core challenge is introducing a representative, narrow sample plug into the separation column with minimal band broadening and without compromising the system’s low volumetric flow rates [30]. In capillary and Nano-LC, where flow rates are in the microliter to nL/min range, even minor dead volumes in the injection pathway can significantly degrade chromatographic resolution [40].

The development of robust, precise, and low-dispersion sample introduction techniques is essential for advancing Mini-LC applications in EOC analysis. Several injection strategies have been developed to meet these demands. A widely adopted solution is the use of integrated trap columns in a “capture-and-elute” configuration [41]. This approach allows for the loading of a relatively large sample volume (e.g., up to 100 µL) onto a trap column at a higher flow rate. Subsequent valve switching directs the mobile phase to flush the concentrated analytes from the trap column onto the analytical column for separation. This method effectively addresses the injection volume mismatch in Nano-LC, pre-concentrates analytes to enhance sensitivity, and enables the on-line desalting or purification of complex samples like food and environmental water extracts or biological fluids. For instance, in the analysis of emerging organic micropollutants in river water, offline SPE is often used as a preliminary concentration and cleanup step, with the final extract being reconstituted in a small volume compatible with Mini-LC injection [42]. Moreover, advancements in microfluidic Chip-LC systems represent the pinnacle of integration. These chips seamlessly incorporate nano-scale injection valves and sample loops directly onto the device, drastically reducing dead volume and ensuring the sample plug is introduced with high precision [43]. The evolution towards these fully integrated, automated injection systems is critical for enhancing the reproducibility, robustness, and analytical performance of Mini-LC methods in the routine monitoring of emerging contaminants. Future trends focus on integrating nanomaterials (e.g., graphene oxide, carbon nanotubes) and bioaffinity-based extraction methods (e.g., immunosorbents, aptamers) to improve selectivity and detection limits. Overall, the development of efficient, green, and high-throughput sample preparation techniques is crucial to unlocking the full potential of Mini-LC in EOC analysis.

### 3.1. Challenges of Sample Preparation for Mini-LC

A key element of Mini-LC workflows is the introduction of samples with low dispersion. Due to the production of very small peak volumes by narrow-bore columns, even slight dead volumes in valves, connectors or injection loops can greatly diminish chromatographic efficiency [44]. Of the available strategies, trap-and-elute setups have emerged as the most commonly used for EOC analysis. A compact trap column enables several microlitres of sample to be introduced at elevated flow rates, followed by valve switching and backflushing into the analytical column [15]. This method addresses the discrepancy in injection volume, improves sensitivity and offers online desalting or purification for complex matrices such as food extracts, wastewater and biological fluids. In river water analysis, solid-phase extraction (SPE) is often performed offline to minimize sample volume, with final reconstitution occurring in a solvent suitable for Mini-LC prior to trap-and-elute injection [42]. In Mini-LC, the injection method must provide a precise, narrow sample plug for narrow-bore columns; usual injection volumes are just a few nanolitres. Larger injections lead to instant band widening and reduced efficiency. Due to the very low peak volumes in nano-LC, even minor dead volumes in valves, loops or fittings can disrupt analyte focusing. Consequently, efficient Mini-LC injection systems depend on low-dispersion components and techniques that focus analytes at the column entrance immediately after introduction.

Several injection methods have been developed to overcome the stringent volume and dispersion constraints of Mini-LC. The choice of method is influenced by the intricacy of the matrix, the required sensitivity and the configuration of the system. Micro-volume rotary valves with loops ranging from 10 nL to 1 µL provide an easy method for delivering small, precisely defined volumes. Mazzoni et al. [45] developed an online SPE/UHPLC-MS/MS method for determining perfluoroalkyl acids in drinking and surface waters. The extraction-to-analysis process took 20 min, and the validated method was used to determine PFFA in waters collected from various locations in Italy. The trap-and-elute setup is the most commonly employed Mini-LC injection method for complex matrices. A short trap column retains analytes while large-volume samples are loaded, allowing unretained matrix elements to be flushed away. Following the valve switch, the analytes are released in a tight band onto the analytical column. This method increases sensitivity, enhances matrix clean-up and reduces extra-column dispersion, making it particularly beneficial for trace-level endocrine-disrupting compound (EDC) analysis in food and environmental samples. Recently, Zhao et al. [46] developed a hybrid monolithic in-tube SPME-UPLC-MS/MS method for the quantification of cannabinoids. This method successfully quantified ten cannabinoids in human urine samples and sewage water, achieving good recovery rates (85.5–112%).

### 3.2. Considerations for Specific Matrices in EOC Analysis

The choice of sample introduction method for Mini-LC is heavily influenced by the complexity of the matrix. For clean aqueous samples such as surface water or wastewater, trap-LC is particularly efficient as it allows direct loading of several microlitres while concentrating trace EOCs and removing particulates. More intricate matrices, such as urine, plasma and various biological fluids, typically require offline dilution or protein precipitation prior to online trapping. In this case, the trap column serves as both a preconcentration unit and a safeguard for the analytical column. Similarly, food and environmental samples benefit from offline solid-phase extraction (SPE), followed by trap-LC, to achieve final purification and analyte concentration. In summary, the progression from basic nano-loop injections to combined trap-and-elute and chip-based technologies has enabled Mini-LC to process more complex samples. Ongoing improvements in automation and minimized dead volumes are anticipated to further enhance performance [45].

## 4. Mini-LC Systems

EOCs are a growing class of compounds that are being separated and detected in various samples, attracting significant attention. These contaminants include pesticides, pharmaceuticals, persistent organic pollutants, perfluoroalkyl substances (PFASs), secondary metabolite contaminants and synthetic organic compounds. Recent advances in Mini-LC systems combined with low-resolution (ion trap or triple quadrupole) or high-resolution (Q-Exactive or Q-TOF) MS have begun to shed light on the distribution patterns and potential health risks associated with these substances.

To provide a clearer overview, the main differences between conventional LC and Mini-LC should be listed in terms of their applications. While both methods share similar separation principles, they differ in terms of column size, operational scale, solvent use, sensitivity, and practical issues related to instrument design and method development. These differences are important because they affect the effectiveness of the analysis, its environmental impact, and its suitability for routine or specific analyses. Table S1 provides a comparison of the two systems’ main features, highlighting their respective advantages and disadvantages. Mini-LC is much easier to use, and both the instrumentation and the connections are operated in the same way as conventional scale LC systems. This opens up opportunities to apply Mini-LC to novel workflows and applications [47]. Despite the advantages of Mini-LC techniques, some limitations must be acknowledged to provide a balanced perspective. For laboratories that use conventional LC techniques, it may be challenging to switch to miniaturized methods that are incompatible with their current protocols [48]. Although Mini-LC systems utilize less solvent and incur lower operational costs, the initial expense of specialized components such as microfabricated columns, compact pumps and high-precision interfaces may exceed that of conventional LC systems. These small parts can also be more difficult to maintain from a technical point of view [49]. Mini-LC systems often present greater experimental challenges, particularly during the various stages of method development and optimization. Because the columns are smaller and have less dead volume, the system is more sensitive to small changes in sample handling, gradient accuracy, and temperature control. Achieving reliable and reproducible separations with Mini-LC techniques may require a higher level of technical skill [50]. When selecting Mini-LC systems for routine or high-throughput applications, it is essential to consider these practical factors thoroughly. Despite these limitations, however, these systems offer an attractive alternative to conventional LC. Mini-LC systems broaden the scope of LC and open up new frontiers, such as drug testing, cellular structure analysis, and microbiological analysis. These systems not only broaden the scope of LC but also open up new frontiers, such as drug testing, the analysis of cellular structures, and microbiologic analyses [51]. There are several types of Mini-LC system, including Cap-LC, Nano-LC, Chip-LC and portable LC systems. These systems offer an attractive alternative to conventional LC. Recent advances have enabled the development of portable systems and online sample processing platforms, significantly contributing to sample monitoring.

Cap-LC, Nano-LC and Chip-LC are three commonly used techniques of Mini-LC. Each has its own advantages and disadvantages. Cap-LC strikes a good balance between size and performance. It features intermediate column diameters and low flow rates (µL min^−1^), which make separation more efficient and require less solvent than conventional LC. It is also mechanically stable and reproducible, providing detection limits in the nanogram range. Nano-LC takes miniaturization much further by utilizing narrow-bore columns and flow rates of nL min^−1^. This makes it highly sensitive down to the picogram level, which is ideal for omics studies and ultra-trace analyses. However, this higher sensitivity can lead to operational issues such as column clogging, flow instability and greater variability in retention time and peak area, making it less suitable for high-throughput analyses. Chip-LC, on the other hand, features a built-in microfabricated chip design that integrates chromatographic channels and injectors into a compact structure. This results in an extremely low dead volume, requires less sample and solvent, and enables use in portable or point-of-care settings. While Chip-LC has many advantages, it is often limited by tolerances in chip manufacturing, reduced process flexibility, shorter channel lengths and significant variability from chip to chip. The lack of standard designs for different brands exacerbates this issue. In summary, Cap-LC prioritizes operational robustness, Nano-LC prioritizes sensitivity at the expense of reproducibility, and Chip-LC prioritizes integration and miniaturization, but struggles with standardization and batch-to-batch consistency.

These techniques differ substantially in terms of their columns, flow rates, sample volumes, separation efficiencies and detection limits. The performance parameters of Cap-LC, Nano-LC and Chip-LC systems are summarized in Table S2.

### 4.1. Cap-LC

**Figure 1:**
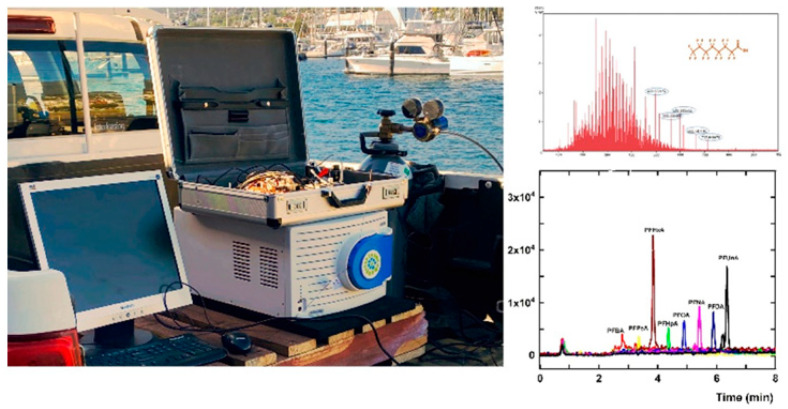
A robust, portable and miniature battery powered gradient Cap-LC system for pharmaceutical analysis (**left panel**) and On-site PFAS analysis (**right panel**). Reproduced with permission [55].

Cap-LC is used to analyze emerging organic contaminants. The main benefit of Cap-LC is that it can separate and analyze compounds at lower concentrations more accurately. This system uses smaller column diameters (e.g., 0.2–1.0 mm) and lower mobile phase volumes. This not only makes the analysis more sensitive, but it also cuts down on waste generation [48,52]. These systems make analyses more efficient and more sensitive to mass by reducing dispersion and using less solvent. This feature is particularly beneficial for analyzing EOCs that contain various organic pollutants [48]. The hyphenation of Cap-LC and mass spectrometry (LC-MS) enables precise detection of organic micro-pollutants, providing high-resolution data that is crucial for environmental monitoring and safety assessments [53]. Cap-LC combined with sensitive detection methods, especially mass spectrometry, has improved detection limits for EOCs, enabling the analysis of trace levels in complex environmental samples. Improvements in micro-flow-controlled techniques also help Cap-LC work faster by making it more sensitive to changes in concentration. Cap-LC provides several unique benefits and is a “greener” technique that uses significantly less mobile phase and is highly flexible and useful in various fields, including EOCs analysis. A portable Cap-LC system were developed to test pharmaceuticals, showing this miniaturized tool could be used in advanced analyses [54]. This flexibility is important for dealing with EOCs that might come from drugs or runoff from farms. Figure 1 shows a field portable Cap-LC/MS system for field based applications [55].

### 4.2. Nano-LC

**Figure 2:**
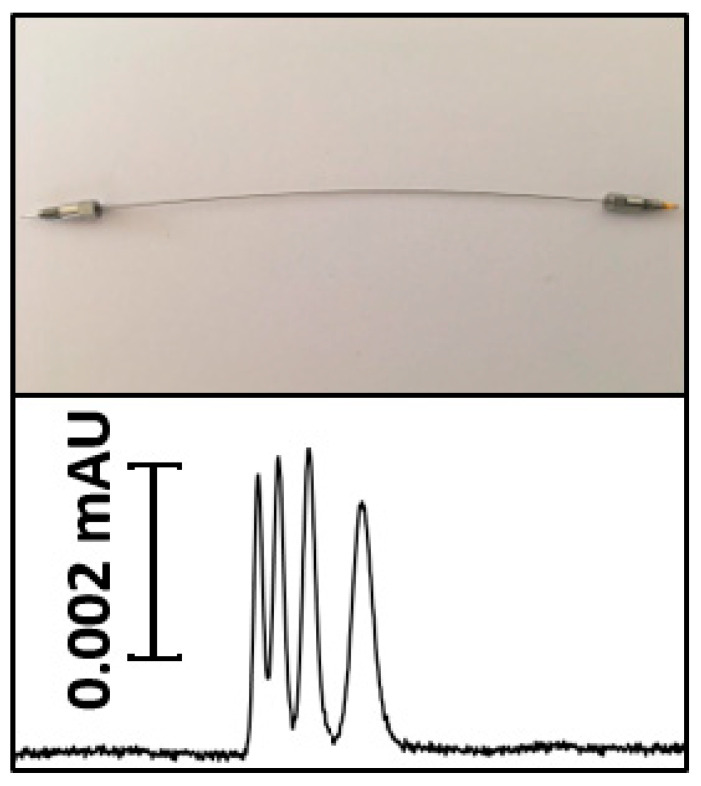
A new metal tubing confined monolithic nano-column with an internal diameter of 20 µm [57].

Nano-LC is a very high sensitive technique for the separation and analysis of different compounds in complex matrices [39]. This technique has profoundly impacted the analysis of organic contaminants, especially as regulatory frameworks increasingly prioritize the detection of pollutants at trace levels. Nano-LC (column i.d. 0.2–0.05 mm, flow rates 2–0.1 μL min^−1^), presents new opportunities to enhance both analytical performance and sustainability [7]. This miniaturization improves chromatographic performance by reducing the inner diameter of the column and flow rates. It also offers substantial environmental and economic benefits, primarily due to a significant reduction in solvent consumption. Several types of column are used in Nano-LC [10]. These columns, including particle packed columns and monolithic columns provide great sensitivity and separation compatibility [12]. The high compatibility of Nano-LC with mass spectrometry significantly amplifies the capabilities of contaminant analysis. This combination promotes high-resolution detection and characterizes the chemical nature of organic contaminants efficiently. Zacs et al. constructed an innovative Nano-LC/MS system for analyzing perfluoroalkyl substances (PFASs) in complex matrices. They also emphasized the importance of overcoming chromatographic peak constraints to improve analytical precision [56]. A new metal tubing confined monolithic nano-column with an internal diameter of 20 µm has been developed for bioanalytical separations in nano-LC. The results in a robust, highly sensitive platform for Nano-LC separations of complex biomolecular samples, offering a promising alternative to existing miniaturized stationary phases in advanced omics applications (see Figure 2) [57]. This encompasses microarray bioassays and MS of components from minute amounts of samples after Nano-LC separation. Nano-LC are used not only for the monitoring of food safety [58,59] but also to monitor the environment, especially to check the quality and safety of water [60]. This adaptation is shown by the selective analysis of different pollutants in water samples. Integrating Nano-LC with advanced materials enhances our ability to detect and remediate complex organic pollutants in various ecosystems.

### 4.3. Chip-Liquid Chromatography

**Figure 3:**
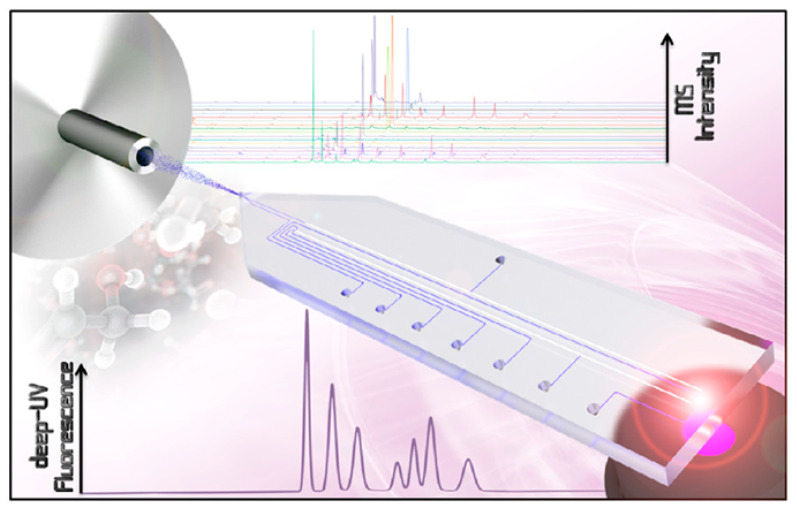
A two-dimensional chip for multiple transfers from the first-dimension effluent onto the column head of the second separation dimension. Reproduced with permission [66].

Fast, modular Chip-LC systems are attracting a great deal of attention for on-line analysis, as they reduce overall solvent consumption by over 80% compared to conventional LC [61,62]. The incorporation of Chip-LC in the analysis of EOCs received a significant progression in modern chromatography [63], mainly owing to its compact design, solvent efficiency, and versatility in handling complex sample matrices. Chip-LC uses microfabrication technologies to make small, efficient separation systems that obtain high-resolution results much faster than regular LC [62]. New microfabrication methods have given Chip-LC technology a significant boost by making it possible to create tiny fluidic channels and work with small sample sizes. Chip-LC technology has a wide range of applications, particularly in fields such as environmental and food safety, where precise contaminant detection is essential [64]. One possible application is the use of a modular, chip-based supercritical fluid chromatography system combined with ambient pressure ionization. This method can quickly separate samples, which is important for meeting the requirements of emerging contaminant analysis [65]. Chip-LC operates in a similar way to regular LC but is integrated into microfluidic designs. Its smaller dimensions make it easy to quickly separate and analyze small samples without compromising sensitivity or resolution. Each design has its own flow characteristics and efficiencies [66]. Figure 3 illustrates a novel, two-dimensional, chip-based-HPLC (2D-chip-HPLC) approach. This method facilitates multiple transfers from the effluent of the first dimension onto the column head of the second separation dimension. This approach is particularly valuable for studying EOCs, as obtaining samples can be challenging due to environmental concerns, as it enables working with small sample volumes. Recent advancements include the integration of Chip-LC with sophisticated detection techniques, such as mass spectrometry and ion mobility spectrometry. For instance, the combination of 2D-chip-HPLC and ion mobility spectrometry (IMS) has revealed significant differences in the separation mechanisms, facilitating the identification and measurement of complex mixtures of contaminants [67]. This combination allows analysts to distinguish compounds based on their size, shape and charge, significantly enhancing the depth of analysis of intricate environmental samples contaminated with various organic pollutants. Furthermore, combining high-resolution mass spectrometry with Chip-LC makes it possible to perform non-targeted analyses, which are crucial for identifying EOCs in complex biological matrices. The combination of Chip-LC and mass spectrometry is increasingly recognized as an effective way of improving the specificity and sensitivity of detection methods. For the structural design and assembly of a polymeric microfluidic chip intended for Chip-LC applications.

## 5. Recent Applications of EOCs in Food and Environmental Samples

There has been an increased awareness towards the use of Mini-LC techniques for the analysis of EOCs for the evaluation of different food samples. Traditional analytical techniques are not suitable for determining food contaminants because they are destructive and time-consuming [68]. Therefore, this section discusses recent and selected analyses of food contaminants by Mini-LC. Readers are also referred to the following review articles [6,7,15,24,63,69,70,71], which include some earlier applications. Table 1 shows the results of all contaminant analyses performed using Mini-LC, alongside several instrumental parameters. 

The assurance of food safety is a paramount global concern, necessitating highly sensitive and selective analytical methods to monitor a wide array of chemical contaminants. These substances, which include mycotoxins, pesticide residues, veterinary drugs, and environmental pollutants, can be present at trace levels in complex food matrices, posing significant risks to human health. The application of Mini-LC, particularly Nano-LC, has proven highly effective for the sensitive determination of various food contaminants [5], including mycotoxins, veterinary drug residues, and pesticides. The ability to achieve low ngL^−1^ or even pgL^−1^ detection limits is crucial for ensuring food safety, as many of these contaminants are toxic even at trace levels. Recent studies have demonstrated that Mini-LC methods can achieve sensitivity levels consistent with international standards (e.g., EU MRLs, US EPA and WHO guidelines) for contaminants such as PFAS, pesticide residues and pharmaceuticals, thereby aligning with regulatory practices. Capillary and microchip LC systems, for example, have successfully quantified pesticide residues at concentrations below EU Maximum Residue Limits (MRLs) and PFAS compounds within U.S. EPA advisory levels. These results confirm that Mini-LC platforms can meet current regulatory expectations for EOC detection and support harmonized monitoring approaches across different jurisdictions.

**Figure 4:**
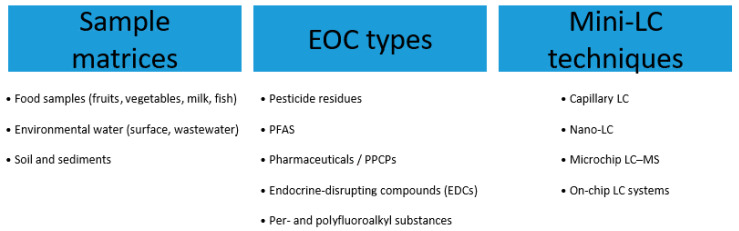
The Mini-LC techniques for the analysis of environmental and food samples for EOCs.

Mini-LC, particularly when coupled with MS, has emerged as a powerful technique in this field. Its exceptional sensitivity, low solvent consumption, and ability to handle complex samples make it ideally suited for the detection and quantification of food contaminants at concentrations often mandated by stringent regulatory limits. Figure 4 summarizes the Mini-LC techniques for the analysis of environmental and food samples for EOCs. With regard to miniaturized-LC-based analyses of food contaminants, emphasis has been placed on articles published since 2022, as previous review articles have focused on earlier periods [5,6,7,24,71]. This section therefore details recent advances and applications of Mini-LC for analyzing key classes of food and environmental EOCs, primarily from early-2022 to mid-2025.

### 5.1. Pesticide Residues

The monitoring of pesticide residues is crucial for assessing compliance with Maximum Residue Levels (MRLs). The trend towards multi-residue methods (MRMs), which aim to analyze hundreds of compounds in a single run, demands high chromatographic resolution. The use of low flowrate not only increases ionization efficiency and minimizes ionization suppression but also boost sensitivity compared to analytical-scale LC–MS methods [58]. Microflow-LC has experienced rapid growth, driven by the emergence of mass spectrometry applications that meet the need for analyzing small volumes of precious samples with ever-higher sensitivity [72]. Cap-LC with column internal diameters of 0.2 to 0.5 mm offers a robust compromise between sensitivity and throughput. The use of long columns packed with sub-2-µm or core–shell particles provides the high peak capacity needed to separate complex mixtures of pesticides and their metabolites. This is especially important for distinguishing between isomeric compounds that co-elute in conventional HPLC. Various column types with different IDs were used to analyze pesticides in given samples [58,73,74,75]. Mini-LC is particularly valuable for analyzing new pesticides with complex compositions. For example, the analysis of short-chain chlorinated paraffins (SCCPs), which consist of thousands of congeners, is nearly impossible with traditional-LC without severe chromatographic interference [76]. The superior efficiency of narrow bore columns, especially when coupled with high-resolution mass spectrometry (HRMS), enables a more detailed congener-specific separation, leading to more accurate quantification. The drastic reduction in organic solvent consumption (often >95% compared to HPLC) aligns with the principles of green chemistry, making Mini-LC an environmentally friendly choice for routine pesticide monitoring laboratories. Various advanced Mini-LC techniques, such as the two-dimensional LC method, were used for pesticide detection in corn products [77]. These techniques are powerful and ‘greener’ alternatives to conventional methods.

### 5.2. Per- and Polyfluoroalkyl Substances (PFAS)

PFAS are a diverse group of synthetic chemicals that have been widely used for over 60 years due to their unique properties. They are persistent EOCs that pose significant health risks through dietary exposure [78]. Despite their widespread use, concerns about the environmental and health impacts of these substances have only recently emerged. These compounds have been detected in several foods, including fish and shellfish, as well as animal-origin foods such as livestock, poultry products and milk. They have also been found in plant-based foods [79]. A Nano-LC/HRMS system, an efficient Mini-LC technique for the analysis of PFAS, was used to analyze PFAS in food products ranging from 0.001 to 0.3 ng g^−1^ [56] and aqueous film-forming foams (AFFFs) and municipal wastewater samples ranging from 0.05 to 0.4 µg g^−1^ [80]. These results are promising for the determination of EOCs with low content in the samples. A sensitive method based on Cap-LC/UV was developed to assess the presence and dissipation of herbicides with a wide range of polarities in soil, using in-tube solid-phase microextraction (IT-SPME) [81]. This study used tritosulfuron (TRT), triflusulfuron-methyl (TRF), aclonifen (ACL) and bifenox (BF) as probe compounds. Suitable linearity was achieved at concentration levels of 0.5–4.0 µg g^−1^ for TRT and TRF, and 0.2–1.0 µg g^−1^ for ACL and BF. Intra- and interday precision (expressed as relative standard deviation) was ≤4% and ≤8%, respectively. The limits of detection (LODs) and quantification (LOQs) were in the ranges 0.05–0.1 µg g^−1^ and 0.1–0.4 µg g^−1^, respectively.

### 5.3. Herbicides

Herbicides (HBs) are chemicals used to kill or inhibit the growth of unwanted plants and weeds. The intensive use of HBs in modern agriculture continues to pose a significant threat to the environment. After application, HBs can disperse to different environmental compartments, such as soil, groundwater or surface water, through various processes. Consequently, many countries have established maximum residue limits (MRLs), specifying limits of no more than 0.1 µg L^−1^ for individual herbicides and 0.5 µg L^−1^ for the cumulative concentration of all herbicides [82]. Therefore, the use of high-sensitivity separation techniques has become a key issue for preventing and controlling HBs. These contaminants were determined using a microfluidic Chip-LC system with 0.0099–0.1388 mmol L^−1^ of the LODs values [82]. Five triazine herbicides were detected in environmental waters using a graphene oxide-ionic liquid-based solid-phase microextraction (SBSE) system coupled to a Cap-LC-MS/MS system, with LOD values ranging from 0.0005 to 0.15 ng mL^−1^ [83]. In addition, the degradation of HBs [84], bifenox and aclonifen, in water was studied using a C18 (150 mm × 0.5 mm ID, 5 µm) column in a Cap-LC/DAD system. This allowed LOD values of 10, 35 and 1550 ng L^−1^ to be obtained for aclonifen (ACL), bifenox (BF) and bifenox acid (BFA), respectively [85].

### 5.4. Veterinary Drug Contaminants

Veterinary drugs, including antibiotics, anthelmintics, and growth promoters, can leave residues in animal-derived products like meat, milk, eggs, and honey. The low flow rates of Mini-LC significantly enhance ionization efficiency in electrospray MS sources, leading to improved signal-to-noise ratios. This high sensitivity is crucial for detecting antibiotic residues such as sulfonamides, tetracyclines, and fluoroquinolones at levels below their respective MRLs [5]. Chloramphenicol (CAP) is an effective broad-spectrum antibiotic against both Gram-positive and Gram-negative bacteria. This was determined using ProFlow Nano-LC/UV with a graphene oxide-functionalized monolithic nano-column, with an LOD of 0.02 µgkg^−1^ [86]. Nano-LC/UV with particle packed column was also used to separate chiral drug contaminants in water samples [87]. This study examined the enantioseparation of ten chiral drugs on CSP-amylose tris(3-chloro-5-methyphenylcarbamate), which was suitable for the coupling of MS detection by dedicated nano-spray interfaces.

### 5.5. Toxins

Marine biotoxins are chemical contaminants that are produced naturally by certain types of algae and other microorganisms, such as bacteria. Ciguatera (CFP) poisoning from eating contaminated fish is the most common type of food poisoning caused by marine biotoxins worldwide, with an estimated 20,000–50,000 cases occurring each year. Cap-LC/HRMS was used to analyze CFP toxins in fish samples, confirming C-CTX1 as the main ciguatoxin present [88,89].

Mycotoxins, toxic secondary metabolites produced by fungi, are among the most pervasive and hazardous food contaminants due to their carcinogenic, teratogenic, and immunosuppressive effects. Their analysis is challenging because of their low abundance in complex matrices like grains, nuts, and spices. The high mass sensitivity of Cap-/Nano-LC [90] and chip-based LC systems [64] is a critical advantage for mycotoxin analysis. For instance, aflatoxins (e.g., Aflatoxin B1, a potent carcinogen) have been successfully determined in nuts and cereals using microfluidic Nano-LC-MS systems [64]. These integrated platforms achieve detection limits as low as 1 ng L^−1^ (ppt), which is essential for compliance with regulatory standards that are often set in the low µg kg^−1^ (ppb) range. The integration of sample preparation and separation on a single chip minimizes sample handling and reduces analyte loss, leading to superior reproducibility. The trap-column approach is frequently employed. A large volume of a purified food extract can be loaded onto a trap column, concentrating the target mycotoxins and allowing for further matrix clean-up online before elution to the analytical column [91]. This is particularly useful for multi-mycotoxin methods targeting groups like aflatoxins, ochratoxin A, and fumonisins simultaneously.

### 5.6. Secondary Metabolite Contaminants

Pyrrolizidine alkaloids (PAs) are chemical compounds naturally produced by some plants and are known for their toxic effects. They can be particularly damaging to the liver, and some are carcinogenic. The advantages of the developed Mini-LC/MS methods include increased sensitivity compared to conventional flow LC-MS. These systems are promising for the sensitive detection of PAs in samples [92].

**Figure 5:**
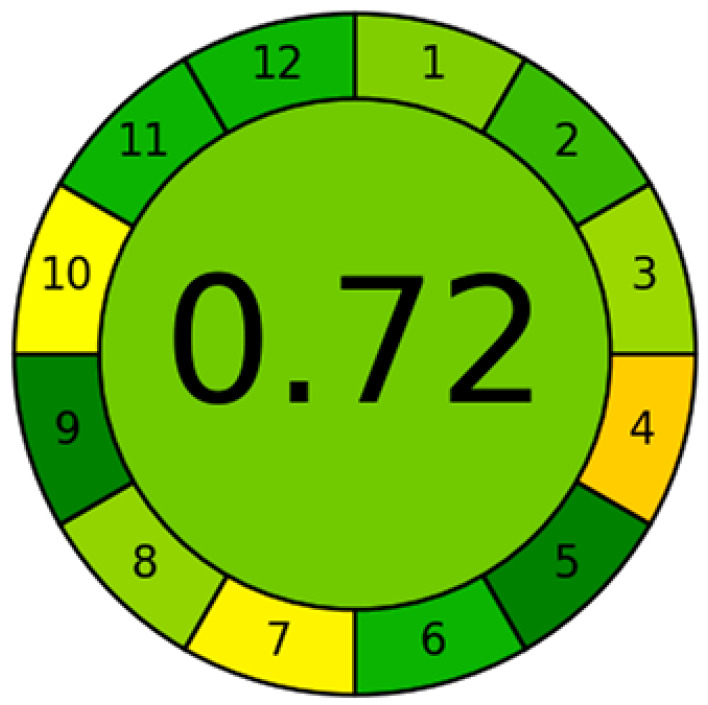
Results of AGREE analysis for the Nano-LC method for Simultaneous Analysis of L-Carnitine and Acetyl-L-Carnitine in food samples. Reproduced with permission [95].

*Garcinia mangostana* L. is widely recognized for its traditional medicinal uses, as well as its growing relevance in the nutraceutical sector. The cytotoxic effects of an alcoholic extract of *Garcinia mangostana* L. were investigated using Cap-LC-ESI-QTOF-MS/MS [93]. This approach provided a better understanding of the cytotoxic potential of the extract against leukemic cells and emphasized its selectivity for cancer cells. Cap-LC system was also applied for non-psychoactive cannabinoids identification [94]. This study also included an AGREE analysis to determine the presence of 24 cannabinoids in hemp inflorescence extracts, in accordance with the requirements of Green Analytical Chemistry standards and using an eco-sustainable analytical method (see Figure 5) [95]. The most widely accepted of these metrics is AGREE, which was used to obtain a final score of 0.71 out of 1.00 using the Mini-LC methodology. This shows that Mini-LC is a promising green analytical methodology. It was also reported that the presence of certain plant contaminants is crucial for metabolic syndrome, as determined using a Nano-LC/HRMS system [96].

### 5.7. Other Contaminants

Mini-LC also finds applications in monitoring other process-derived contaminants [97,98]. Certain PAHs, like benzo[a]pyrene, are genotoxic and can form in food during grilling or smoking [99]. Mini-LC provides the necessary sensitivity for the determination of relevant compounds in the samples [97,100,101]. When coupled with MS, it allows for the confident identification and quantification of a broader range of PAHs in complex food matrices like oils and smoked meats. A combination of weir-based blockage and single-particle frit was employed to separate PAHs by passing nano-sized silica particles through a miniaturized glass microchip channel [102]. PAHs were also separated using monolithic columns incorporating carbon dots by RP/Cap-LC [103]. Contaminants such as acrylamide or furan, which form during thermal processing of food, can also be analyzed using Mini-LC-MS/MS. The high sensitivity required for these small, polar molecules in starchy foods like potato chips or coffee is effectively achieved with Cap-LC or Nano-LC systems.

## 6. Technical Challenges and Prospects of Mini-LC

Mini-LC faces some technical challenges that prevent its regular use. A significant issue is matrix interference: the narrow-bore columns and low-volume injection routes are easily affected by proteins, lipids, humic substances and salts, which can contaminate the system or hinder MS detection. Even with trap-and-elute setups, highly complex samples often require extensive pretreatment to ensure consistent performance. The stability of instruments poses another challenge. Working with nano- and micro- flow rates requires precise pump, valve, and temperature regulation, as minor variations can significantly affect retention time and peak shape. The restricted loading capacity of Mini-LC columns adds to the complexity of analysis as they are more prone to overload and can degrade rapidly when subjected to concentrated or inadequately purified extracts. Furthermore, the lack of uniformity in column formats, interfaces and operating protocols across manufacturers complicates method transfer and extensive method validation. Despite these difficulties, the outlook for Mini-LC remains positive. Progress in microfluidics, enhanced low-flow pump technologies, and stronger trapping and extraction materials continually improves system reliability and user-friendliness. Enhanced standardization and integration of sample preparation with Mini-LC, especially in narrow-column and chip-based platforms, is expected to improve accessibility and facilitate the wider use of regular monitoring of emerging contaminants.

## 7. Conclusions

Mini-LC has firmly established itself as one of the most powerful and sustainable analytical approaches for determining emerging organic contaminants (EOCs) in food and environmental samples. Continued miniaturization of chromatographic systems encompassing capillary-, nano- and chip-LC configurations—has substantially improved separation efficiency, sensitivity and eco-efficiency by minimizing solvent use and adopting a compact design. Coupling these systems with high-resolution mass spectrometry enables the reliable detection of EOCs at trace and ultra-trace levels across complex matrices, thereby advancing both food safety and environmental monitoring. Nevertheless, several technical and methodological challenges remain. These include issues related to column standardization, matrix effects, reduced injection capacities and the robustness of low-flow systems. Addressing these issues is essential to facilitate broader routine applications. Progress in microfabrication, robust low-volume pumps and intelligent fluid handling is expected to further improve method reproducibility and portability.

Looking to the future, the emergence of miniaturized two-dimensional liquid chromatography (2D-LC) offers a promising way to enhance peak capacity and multidimensional separation in micro- and nanoscale formats. The development of lab-on-chip mini-LC devices with fully integrated sample preparation, separation and detection modules could transform on-site monitoring by providing a scalable platform for real-time environmental diagnostics and rapid food contamination screening. Furthermore, integration with artificial intelligence (AI) and machine learning algorithms will facilitate the automated optimization of chromatographic parameters, intelligent peak deconvolution and predictive data analytics. This will lead to faster method development and more accurate contaminant classification. In summary, the convergence of advanced materials, microfluidic engineering and AI-based data processing will define the next generation of Mini-LC systems. It is anticipated that these future developments will transform Mini-LC from a specialized research tool into a standardized, autonomous and field-deployable technology for comprehensive EOC monitoring and sustainable analytical practice.

## Figures and Tables

**Table 1:** EOC analyses performed using Mini-LC techniques, alongside some instrumental parameters.

Analytical System with Detector	Analyte	Matrix	LOQ (µg kg^−1^)	Ref.
Nano-LC/HRMS	Pesticide	Food	0.01	68
Micro LC-MS/MS	Pesticide	-	-	70
Micro LC-MS/MS	Pesticide	Fruits and Vegetables	10	75
Open-tubular Nano-LC/LRMS	Pesticide	-		76
Micro LC/HRMS	Chlorinated paraffins	Infant milk	-	77
2D-Micro LC-MS/MS	Pesticide	Corn products	7.2	78
Nano-LC/HRMS	PFAS	Foods products	0.02–0.05	79
Nano-LC/HRMS	PFAS	Wastewater	-	80
Cap-LC/UV	Tritosulfuron, triflusulfuron-methyl, aclonifen, and bifenox	Environmental waters	100–400	81
Cap-LC-MS/MS	Phenylurea herbicides	Water	330–462	82
Cap-LC-MS/MS	Triazine herbicides	Cap-LC-MS/MS	-	83
Nano-LC/DAD	Ribenuron methyl	Water	1–5	84
Cap-LC/UV	Diphenyl-Ether Herbicides	Environmental waters	0.01–1.55	85
Nano-LC/UV	Chloramphenicol	Honey	0.08	86
Nano-LC/UV	Chiral drugs	Water	-	87
Cap-LC/HRMS	Ciguatoxin	Fish samples	0.6	88
Cap-LC/HRMS	Ciguatoxin	Fish	0.6–20	89
Cap-LC/UV	Aflatoxin B1 B2 G1 G2	Aqueous samples	-	90
Chip-Nano-LC/LRMS	Aflatoxin	Peanut samples	0.048	91
Nano-LC/HRMS	Ricin	Castor bean extract	-	92
Nano-LC/HRMS	Pyrrolizidine alkaloids	Tea, honey, herbal tinctures, and milk samples	0.33–3.6	93
Nano-LC/DAD	Chlorophylls a and b and β-carotene	Environmental waters	0.3–1.5	97
Nano-LC/UV	alkylphenols	Tea samples	-	98
Cap-LC/LRMS	Haloacetic acids	Municipal tap water and swimming pool water	9.3–71.4	100
Microchip-LC/LRMS	Biogenic amines	-	0.156–3.11	101
Microchip-LC/UV	PAHs	River water	0.002–0.04	102

## Data Availability

No new data were created or analyzed in this study. Data sharing is not applicable to this article.

## Supplementary Materials

The following supporting information can be downloaded at: https://www.mdpi.com/article/10.3390/molecules31010068/s1, Table S1: Comparison of miniaturized sample preparation techniques for Mini-LC analysis of EOCs; Table S2: The key features are compared in terms of the advantages and disadvantages of conventional LC and Mini-LC systems; Table S3: Performance Parameters of Cap-LC, Nano-LC, and Chip-LC Systems.
