# Supplementary material for: Miniaturized-LC in the Analysis of Emerging Organic Contaminants in Food and Environmental Samples: Recent Advances and Applications
Source: Molecules. 2025 Dec 24;31(1):68. doi: 10.3390/molecules31010068 (PMC12787042; doi:10.3390/molecules31010068)
Supplement: Supplementary file 1 [file molecules-31-00068-s001.zip › molecules-3991433-supplementary.pdf]

Supplementary Information

Miniaturized-LC in the Analysis of Emerging Organic Contaminants in Food and Environmental Samples: Recent Advances and Applications

Cemil Aydoğan [1,2,3,*], Ashraf Ali [4], Mehmet Atakay [5], Bekir Salih [5,6] and Ziad El Rassi [7,*]

[1] Food Analysis and Research Laboratory, Bingöl University, 12000 Bingöl, Türkiye
[2] Department of Chemistry, Bingöl University, 12000 Bingöl, Türkiye
[3] Department of Food Engineering, Bingöl University, 12000 Bingöl, Türkiye
[4] School of Chemistry and Chemical Engineering, Henan University of Technology, Zhengzhou 450000, China; ashrafchemist12@gmail.com
[5] Department of Chemistry, Hacettepe University, 06800 Ankara, Türkiye; mehmetatakay@hacettepe.edu.tr (M.A.); bekir@hacettepe.edu.tr (B.S.)
[6] Turkish Academy of Sciences, 06670 Ankara, Türkiye
[7] Department of Chemistry, Oklahoma State University, Stillwater, OK 74078-3071, USA

*Corresponding author : caydogan@bingol.edu.tr (C.A.); elrassi@okstate.edu (Z.E.R.)
Fax: +90 426 216 00 33 （C.A.）

ORCIDs:

Cemil AYDOĞAN: https://orcid.org/0000-0002-7692-0423

Table S1. Comparison of miniaturized sample preparation techniques for Mini-LC analysis of EOCs.

Technique	Principle	Matrices	Advantages	Limitations	Reference
µ-SPE / IT-SPME	Adsorption on selective micro-sorbents (C18, HLB, MIPs)	Environmental water, food extracts, plasma	Low solvent use; compatible with direct Mini-LC coupling; high enrichment	Limited loading capacity; sorbent fouling	[36]
LLME	Dispersive extraction using micro-volume extractants	Waters, beverages	Very high enrichment; rapid	Less suitable for oily/solid matrices; requires clean-up	[37]
QuEChERS	Salt-assisted extraction + d-SPE	Food matrices	Multi-residue capability; good for pesticides, toxins	Extracts often require additional cleanup for Mini-LC	[38]
On-line Trap-and-Elute	Concentration on trap column, back-flush to analytical column	All matrices (especially waters & biological samples)	Excellent sensitivity; eliminates volume mismatch	Requires specialized valves; pressure compatibility	[45]

Table S2. The key features are compared in terms of the advantages and disadvantages of conventional LC and Mini-LC systems.

Feature	Conventional LC	Miniaturized LC
**Column dimensions**	Larger internal diameter (2.1-4.6 mm), longer columns	Narrow internal diameter (<1 mm), shorter columns, micro/nano-scale systems
**Flow rate**	Typically, 0.2-1.5 mL/min	Significantly reduced (nL/min-μL/min)
**Solvent consumption**	High solvent usage, high operating cost, and waste generation	Very low solvent consumption; environmentally friendly and cost-efficient
**Sample volume requirement**	Requires larger sample volumes (μL-mL)	Compatible with very small sample volumes (nL-μL)
**Separation efficiency**	Good efficiency but limited by mass-transfer constraints	Higher efficiency due to reduced diffusion path and improved mass transfer
**Sensitivity**	Moderate sensitivity: sample dilution can occur	Higher sensitivity due to reduced band broadening and lower flow rates
**Instrument**	Bulky, benchtop systems	Compact or portable systems, suitable for field applications
**Operating**	Well-established workflows; user-friendly for routine labs	More technically demanding; requires expertise in handling low-dead-volume systems
**Cost considerations**	Lower initial instrument cost but higher solvent/maintenance costs	Higher initial cost for specialized components; lower long-term solvent cost
**Method development**	Robust and widely standardized; easier optimization	More sensitive to system dead volume, temperature, and gradient precision
**Standardization**	Highly standardized across the industry	Limited standardization; variability between manufacturers
**Routine adoption**	Widely used in QC, pharmaceutical, clinical, and research labs	Adoption limited; growing interest in specialized or micro-scale applications
**Environmental impact**	Larger solvent waste	Significantly reduced environmental footprint

Table S3. Performance Parameters of Cap-LC, Nano-LC, and Chip-LC Systems.

Parameter	Cap-LC	Nano-LC	Chip-LC
**Column inner diameter**	150-500 µm	50-100 µm	Integrated microchannels (10-75 µm equivalent)
**Typical flow rate**	1-10 µL/min	50-500 nL/min	nL/min to low µL/min (chip-dependent)
**Sample volume requirement**	Low (µL scale)	Very low (nL-µL scale)	Very low (nL-µL; integrated injection)
**Separation efficiency**	High (H < 10 µm)* (20,000 – 100,000 plates)	Very high (improved mass transfer) (100,000 – 500,000 plates)	Moderate-high (chip fabrication-dependent) (10,000 – 150,000 plates)
**Detection limit**	Low to moderate sensitivity (10-500 ng/mL)	Very high sensitivity (10-1000 pg/mL)	High sensitivity due to minimized dead volume; performance varies with chip design (50 pg/mL-1 ng/mL)

*H, "Height equivalent to a theoretical plate"
